# Use of traditional and modern contraceptives among childbearing women: findings from a mixed methods study in two southwestern Nigerian states

**DOI:** 10.1186/s12889-018-5522-6

**Published:** 2018-05-09

**Authors:** Anthony Idowu Ajayi, Oladele Vincent Adeniyi, Wilson Akpan

**Affiliations:** 1 Department of Sociology, Faculty of Social Sciences & Humanities, University of Fort Hare, 50 Church street, East London, 5200 Eastern Cape South Africa; 2 Department of Family Medicine, Cecilia Makiwane Hospital, East London Hospital Complex, Walter Sisulu University, East London, Eastern Cape South Africa

**Keywords:** Contraceptive methods, Family planning, Child spacing, Modern contraception, Traditional contraception, Southwestern Nigeria

## Abstract

**Background:**

Contraceptive use has numerous health benefits such as preventing unplanned pregnancies, ensuring optimum spacing between births, reducing maternal and child mortality, and improving the lives of women and children in general. This study examines the level of contraceptive use, its determinants, reasons for non-use of contraception among women in the reproductive age group (18–49 years) in two southwestern Nigerian states.

**Methods:**

The study adopted an interviewer-administered questionnaire to collect data from 809 participants selected using a 3-stage cluster random sampling technique. We also conducted 46 in-depth interviews. In order to investigate the association between the socio-demographic variables and use of contraceptive methods, we estimated the binary logistic regression models.

**Results:**

The findings indicated that knowledge of any methods of contraception was almost universal among the participants. The rates of ever use and current use of contraception was 80 and 66.6%, respectively. However, only 43.9% of the participants had ever used any modern contraceptive methods, considered to be more reliable. The fear of side effects of modern contraceptive methods drove women to rely on less effective traditional methods (withdrawal and rhythm methods). Some women employed crude and unproven contraceptive methods to prevent pregnancies.

**Conclusion:**

Our findings show that the rate of contraceptive use was high in the study setting. However, many women chose less effective traditional contraceptive methods over more effective modern contraceptive methods due to fear of side effects of the latter. Patient education on the various options of modern contraceptives, their side effects and management would be crucial towards expanding the family planning services in the study setting.

## Background

Contraceptive use has numerous health benefits such as preventing unplanned pregnancies, ensuring optimum spacing between births, reducing maternal and child mortality, and improving the lives of women and children in general [1–6]. Recent estimates show 54 million unplanned pregnancies, 79,000 maternal deaths and 1.2million childhood mortality could have been prevented with universal access to effective family planning methods [2, 6–8]. Despite the aggressive effort of government and non-governmental agencies to improve access to and use of contraceptive methods in sub-Saharan Africa over the past 3 decades, the dominant studies reported low levels of contraceptive use [9–11].

The most recent Nigeria Demographic and Health Survey (NDHS) data showed that only 14.5% of couples in country reported using any methods of contraception [12]. However, the rate of contraceptive use is much higher in the southwestern region of Nigeria. The NDHS report shows that 38% of women in southwestern Nigeria are using any form of contraceptives [12]. However, the findings from National Bureau of Statistics [13] and other smaller studies in the region suggest that the rate could be higher [14–19]. However, it is important to note that the smaller studies were conducted among specific population (women attending antenatal care services and women residing in rural communities), which may explain their results.

Access to family planning services has significantly been expanded in the southwestern Nigeria over the past 10 years and contraceptive knowledge is almost universal among women. As such, previous estimates of contraceptive use may not reflect the current level of contraceptive use. This at least provides a basis for further studies.

Many studies have reported low use of traditional contraception in Nigeria [11, 14, 16, 20]. Low levels of education, women’s and partners’ disapproval of modern family planning methods, religious beliefs, fear of side effects of modern contraception, women’s misconceptions of contraceptive side effects, use of unproven methods or concoctions and infrequent sex are among the reasons for non-use of contraception in sub-Saharan Africa [10, 21–24]. However, many of the aforementioned reasons relate more to the use of modern contraceptive methods rather than natural or traditional family planning methods. Considering that contraceptive methods include the use of traditional methods such as withdrawal and rhythm methods —even though they are less effective— then many reasons advanced in the literature do not sufficiently explain the reasons for low level of use of any form of contraception and traditional methods, in particular, in sub-Saharan Africa. Early anthropological studies suggest that use of traditional family methods (periodic abstinence) is common in sub-Saharan Africa countries [25–33], especially southwestern Nigeria. Orubuloye [30] notes that postpartum sexual abstinence has probably reduced. Although postpartum abstinence has evidently reduced, the need to space children is still paramount.

It is against this background that this article using a mixed-methods study examines the level of contraceptive use, its determinants, reasons for non-use of contraception among women of the reproductive age group (18–49 years) in South Western Nigeria. This study also begins with the assumption that low level of traditional family planning methods in previous studies may be due to women’s reticence of reporting their contraceptive choices. Recently published studies among women in Malawi and Zimbabwe that show discordance between self-reported contraceptive use and the presence of contraceptive progestins in serum lay credence to our assumption [34, 35]. Also, the cultural sensitivity of discussing sexual issues in the sub-Saharan Africa context and the possible reticence of women to respond to contraceptive questions may explain the possible underestimation of the level of traditional contraceptive use. Blackstone and Iwelunmor’s [11] study indicates that about half of men agreed that women who use contraceptives become promiscuous. Women might not be open to revealing if they are utilising any family planning methods or simply reply negatively to wade off further questioning.

## Methods

### Study settings

The data analysed in this study came from a large study (MANCONFREE), which examined the maternal outcomes in the context of free maternal healthcare in South Western and North Central Nigeria. The study was conducted between May and September 2016. The analysis in this study was limited to women who had given birth in the preceding 5 years of the survey in two South Western Nigeria states— Ondo and Ekiti states. South Western Nigeria is one of the six geopolitical zones of Nigeria and has the highest rate of contraceptive use in Nigeria [12]. The region comprises of six states of the 36 states that make up Nigeria. This study was conducted in two of the six southwestern Nigeria states.

### Sampling design

The study adopted a 3-stage cluster random sampling technique. 809 women took part in the study; the sample size of 809 was based on a confidence interval of ±3.5 adjusted for possible missing responses. The study took place in Ondo and Ekiti states randomly selected from the six South Western Nigerian states. We randomly selected these two states because contraceptive use in southwestern Nigeria states is similar (see NDHS 2013). The 2006 census list of enumeration areas (EAs) was the sample frame used for sample selection. Each state was clustered into enumeration units, and they were stratified based on rural, peri-urban and urban areas. The study took place in 54 enumeration areas. 15 households were randomly selected in each enumeration area. Every 10th household in selected EAs was visited to identify study participants until the sample size of 809 women was reached. We selected every 10th households in order to include newly built houses in each EAs since 2006. Households without women that gave birth during the specified period or with women who refused to participate in the study were skipped. Participants of the in-depth interviews were conveniently selected.

### Data collection

The data collected began with the semi-structured in-depth interviews. 46 IDIs were conducted and the topics covered in each interview include: birth spacing, knowledge of contraception, use of contraception and reasons for non-use of contraception. Each interview lasted for an average of 20 min, and all interviews were tape-recorded and assigned codes. Recruitment of informants and interim analyses were conducted after every 10 interviews until no new information emerged from the interviews (data saturation). The interviews helped in modifying the survey instrument.

Using an interviewer-administered questionnaire, 809 women who met the study criteria completed the questionnaire. The questions in the questionnaire were derived from the NDHS pre-validated questionnaire. We piloted the questionnaire among 20 women who were not included in the main study. The questionnaire comprised of four key sections. The first part comprised of questions probing participants’ demographic characteristics. Here, participants reported their age, level of education, place of residence, ethnic group, marital status, level of income and number of children. Part two of the questionnaire dealt with knowledge and awareness of various contraceptive methods. Here a list of 12 contraceptive methods was described to participants and the researcher asked if participants were aware of each method. The third part probed the practice of contraception. Participants were asked if they had ever used and were currently using any form of contraception and if they respond affirmatively, the specific method ever used and currently in use was probed. For participants that were reticent to respond or who replied negatively, the researcher and his well-trained assistants would ask about the interval of child spacing between participants’ children to establish the practice of birth control. Subsequently, the methods of preventing pregnancy during that interval were probed. For participants that were still coy in responding or that stated that they used knowledge of God, the researcher often probed further by asking the participants to explain how they avoided pregnancy during those intervals. Lastly, each of the methods specified in part one were then described to the women in their native language and the researcher would ask if they currently or had used any of the methods. The last part examined reasons for non-use of contraception and contraceptive discontinuation.

### Statistical analysis

The quantitative data were analysed with Statistical Package for the Social Sciences (SPSS version 21). Frequency distribution and cross-tabulation of variables of interest were computed and significant variables were determined with a *p*-value less than 0.05. Significant variables associated with the use of contraceptive methods were included in the binary logistic regression models. To account for the complex sampling strategy and to adjust for non-response, sampling weights were assigned before performing the analysis. Statistics for complex sample analysis feature of SPSS was used in performing the data analysis.

The qualitative data were analysed using content analysis. Themes were developed after reflexive reading of the transcriptions. All authors cross-validated the instruments and the emerging themes.

### Ethical consideration

The University of Fort Hare’s Research Ethical Committee (UREC) approved the study protocol (AKP031SAJA01). Also, the study protocol was reviewed and approved by the Ondo State Health Research Ethics Committee (OSHREC) [NHREC/18/08/2016]. In addition, permission to implement the study was obtained from community heads and household heads. All the participants signed a written informed consent to indicate their willingness to take part in the study. Voluntary participation, confidentiality and anonymity were strictly observed.

## Results

Participants’ mean age was 31.3 (SD 6.2) and their mean parity was 2.7 (SD =1.4). Most participants were Christians (83.7%), Yoruba (87.5%), married (96.2%), employed (85.5%), owned a mobile phone (94.1%), watched television (95.8%), owned a bank account (57.8%), earned at least ₦20,000 (69.3%) and belonged to middle socioeconomic status (57.2%). Only a few participants (32.1%) had access to Internet (Table 1).

**Table 1:** Demographic characteristics of respondents

Variables	Frequencies (*n* = 809)	Percentages
Age		
20 years and below	30	3.7
21–25	127	15.9
26–30	249	30.8
31–35	199	24.6
36–40	146	18.0
Above 40	58	7.2
Level of education		
No formal education	8	1.0
Primary education	114	14.1
Secondary education	435	53.8
Tertiary education	252	31.1
Place of residence		
Urban	307	37.9
Peri-urban	207	25.6
Rural	295	36.5
Religion		
Christian	677	83.7
Muslim	132	16.3
Ethnic group		
Yoruba	706	87.5
Igbo	45	5.6
Ebira	21	2.6
Others	35	4.2
Marital status		
Currently married	778	96.2
Formerly married	5	0.6
Never married	26	3.2
Employed	692	85.5
Own a phone	761	94.1
Watch television	775	95.8
Own bank account	468	57.8
Use internet	260	32.1
Number of children		
One	189	23.4
Two	200	24.7
Three	206	25.5
Four	141	17.4
Above four	73	8.8
Income categories		
No income	118	14.7
N20000 and below	557	69.3
Above N20000	129	16.0
Socioeconomic status		
Low	52	6.5
Middle	461	57.2
High	293	36.4

### Awareness and knowledge of contraceptive methods

The majority of the participants were aware of both modern and traditional contraceptive methods, except male sterilisation. Most of the participants had heard of the contraceptive methods from the antenatal clinic. Only a few participants were aware of folk methods. The use of locally made potion of cooked leaves and/or a traditional ring and/or alcohol as contraceptive methods were categorised as folk methods. In the IDI phase, participants were asked to mention the name of the oral pills they were aware of, and to explain the rhythm and standard day methods. The findings showed that awareness of a method did not necessarily mean knowledge of that particular method. For instance, some participants had heard about female condoms but had never seen one nor did they understand how to use it. Similarly, some participants gave an inaccurate description of standard day and rhythm methods, and some simply mistook non-emergency contraceptive drugs as emergency contraceptive pills.

### Family planning practice among women in Ondo and Ekiti states

Eliciting information on use of contraception was not particularly straightforward. While some women would give immediate responses, others were shy to disclose whether they had done anything to prevent unplanned pregnancy or not. Most women that use injectables, oral pills, implants and IUDs were more forthcoming in their responses than those that used condoms and withdrawal methods. For those that used the withdrawal method and condoms, they were very shy to disclose and often used pseudo terms to denote withdrawal and condoms. For instance, a middle-aged woman in Ekiti referred to a condom as “ade daddy” (meaning ‘daddy’s crown’) and another young mother in Ondo state referred to a condom as “fila daddy” (meaning ‘daddy’s cap’). Getting many of the participants that were shy to disclose their family planning method required probing with two or more questions and even description of all methods to get participants to identify which method they used.

The majority of the participants (80%) reported ever use of a method of contraception. A few respondents did not reveal whether or not they had used any method of contraception. The combination of periodic abstinence and standard day/Rhythm (31.1%), withdrawal (16.4%), condom (17.2%), oral pills (18.8%), and injectables (10.7%) were the main contraceptive methods ever used by the participants (Table 2). Use of concoctions, non-emergency contraception douching and were reported by a few participants. Of the total participants, only 43.9% had ever used modern contraceptives. Traditional planning methods constituted an important component of contraception practice and they were evidenced in its use by a 32.4% distribution amongst all the participants. Thirty-four participants refused to answer the question for personal reasons.

**Table 2:** Awareness and use of contraceptive methods

Variables	Aware of contraceptive methods (*n* = 809)	Ever use *n* = 645	Current use (*n* = 538)
Male sterilisation	155 (19.5)	0 (0)	0 (0.0)
Female sterilisation	782 (98.6)	2 (0.3)	1 (0.2)
Intrauterine device (IUD)	595 (74.9)	24 (3.7)	22 (4.1)
Injectable	768 (96.7)	66 (10.2)	54 (10.0)
Implants	730 (92.1)	27 (4.2)	23 (4.3)
Oral pills	769 (97.2)	116 (18.0)	72 (13.4)
Male Condom	782 (98.6)	106 (16.4)	96 (17.8)
Female condom	738 (93.1)	1 (0.2)	1 (0.2)
Emergency contraception	708 (89.3)	13 (2.0)	12 (2.2)
Rhythm method/Standard day method	737 (92.9)	144 (22.3)	142 (26.4)
Lactation Amenorrhea	734 (92.6)	7 (1.1)	6 (1.1)
Withdrawal method	748 (94.3)	101 (15.7)	99 (16.8)
Folk methods	48 (6.1)	8 (1.2)	10 (1.8)

Key: folk methods- use of lime, alcohol, salt and water, douching/extraction of sperm and amplicox as emergency contraception

When asked if participants were currently using or doing anything to prevent unplanned pregnancies the majority of the participants (*n* = 538; 66.6%) answered affirmatively. The combination of periodic abstinence and standard day/Rhythm (26.1%), withdrawal (18.4%), condom (17.8%), oral pills (13.4%), and injectables (10.0%) are the contraceptive methods most women were currently using in the study setting. Only 8.6% of the participants currently used long acting contraceptive methods. Of the participants (*n* = 538) that were currently using any form of contraception, 52.2% were using a modern contraceptive method. Oral pills, condom and injectables were the commonly used modern contraceptive methods (Table 2).

### Factors associated with of contraceptive

The proportion of women that had ever used any form of contraception was highest in urban areas (92.8%), among women that had completed higher education (87.2%), were aged above 25 years (87.1%), with two or more children (77.1%), and of high socioeconomic status (79.2%) (Table 3). In the logistic regression, after adjusting for confounding variable (education), only urban residence (AOR: 3.7; CI: 2.2–6.0), peri-urban residence (AOR:2.3; 95% CI:1.4–3.1), and high socioeconomic status (AOR:2.1; CI: 1.01–4.3) were the independent predictors of ever use of contraception. In reference to women who had one child, women with two or more children had higher odds of reporting ever used of any contraceptive methods.

**Table 3:** Factors associated with use of contraceptive

Variables	Ever use any methods (*n* = 645)		Ever used any modern methods (355)		Current use of any contraception methods (538)		Current use of any modern contraceptive methods (281)	
	Frequency (%)	AOR (CI)	Frequency (%)	AOR (CI)	Frequency (%)	AOR (CI)	Frequency (%)	AOR (CI)
Age								
30 and below	305 (79.2)	Ref	174 (43.0)	Ref	251 (65.2)	Ref	138 (34.1)	Ref
Above 30	339 (87.1)	1.3 (0.8–2.0)	181 (44.9)		287 (73.8)	1.0 (0.7–1.5)	143 (35.5)	0.8 (0.6–1.1)
Level of education								
No formal education	4 (50.0)	–	1 (12.5)	–	3 (37.5)		0 (0.0)	–
Primary	86 (79.6)	–	50 (43.9)	–	70 (64.8)		39 (34.2)	–
Secondary	344(82.5)	–	193 (44.4)	–	286 (68.6)		150 (34.5)	–
Higher degree	211 (87.2)	–	111 (44.0)	–	180 (74.4)		92 (36.5)	–
Place of residence								
Urban	270 (92.8)	3.7 (2.2–6.0)***	130 (42.3)	1.6 (1.1–2.2)*	248 (85.2)	4.5 (3.0–6.8)***	122 (39.7)	2.1 (1.4–3.0)***
Peri-urban	167 (83.9)	2.3 (1.4–3.8)**	100 (48.3)	1.7 (1.2–2.5)*	137 (68.8)	2.2 (1.5–3.3)***	77 (37.2)	1.9 (1.3–2.9)***
Rural	208 (73.0)		125 (42.4)		154 (54.0)	Ref	82 (27.8)	Ref
Socioeconomic status								
Low	33 (64.7)	Ref	26 (50.0)	Ref	24 (47.1)	Ref	19 (36.5)	Ref
Middle	361 (82.6)	1.8 (0.9–3.5)	199 (43.2)	1.0 (0.5–1.8)	301 (68.9)	1.7 (0.9–3.2)	152 (33.0)	0.9 (0.5–1.7)
High	248 (87.3)	2.1 (1.01–4.3)*	130 (44.4)	1.2 (0.6–2.3)	212 (74.6)	1.9 (1.0–3.6)	110 (37.5)	1.0 (0.5–2.0)
Parity								
1	128 (73.1)	Ref	64 (33.9)	Ref	102 (58.3)	Ref	52 (27.5)	Ref
2	177 (91.7)	3.9 (2.2–7.1)***	99 (49.5)	1.9 (1.2–2.9)*	150 (77.7)	2.9 (1.8–4.8)***	78 (39.0)	1.6 (1.1–2.5)*
3	165 (82.5)	1.9 (1.1–3.3)*	89 (43.2)	1.4 (0.9–2.2)	142 (71.0)	2.1 (1.3–3.4)***	71 (34.5)	1.4 (0.9–2.2)
4 and above	175 (84.5)	2.7 (1.5–4.8)***	103 (48.9)	1.9 (1.2–3.0)*	145 (70.0)	2.4 (1.5–4.1)***	80 (37.4)	1.7 (1.03–2.7)*

Models adjusted for level of education; *Ref* reference, *AOR* adjusted odd ratio, *CI* confidence interval

*** Means *p* < 001, ** Means *P* < 0.005, * Means *P* < 0.05

In reference to women residing in rural residences, women residing in urban residences (AOR: 4.5; CI: 3.0–6.8) and peri-urban residences (AOR: 2.2; CI:1.5–3.3) were more likely to be current users of any method of contraception (Table 3). Also, women who had two or more children had higher odds of reporting current use of any contraceptive methods compared to women who had one child. Likewise, women who resided in urban areas (AOR:2.1; 95% CI: 1.4–3.0) and peri-urban areas (AOR: 1.9; 95% CI: 1.3–2.9) had higher odds of reporting current use of modern contraception relative to women in rural areas. Compared with women who had one child, women with two children (AOR: 1.6; 95% CI: 1.1–2.5) and four or more children (AOR:1.7; 95% CI:1.03–2.7) had higher odds of reporting current use of a modern contraceptive method.

### Reasons for non-use of contraception

The in-depth interviews provided far-reaching analysis of the dynamics of contraceptive use among South Western Nigerian women. The reasons for non-use of contraception is arranged and discussed under the following themes:

### Fear of side effects

The fear of side effects of modern contraception was the most reported reason for non-use of contraception and modern contraception in particular. Some women believe that modern contraceptives are harmful to the body and as such fails to use contraceptives or rely on less effective traditional family planning methods. The response of a 23-year-old mother of 2 children who had a secondary level of education epitomised this view:

“*I do not use pills or condoms because I am afraid of their side effects” (IDI Participant 1, 12 July 2017 Ekiti state*).

In a bid to still prevent pregnancy, some women practice some unconventional and unreliable methods. For instance a woman described what she does to prevent pregnancy by stating: *“I use the withdrawal method and sometimes maintain top position during sex to hinder the flow of sperm. I am scared of using modern family planning methods”.* Maintain top position during sex may not prevent a woman from getting pregnant. However, this underscores that unmet need for family planning is not limited to lack of access, but also because of perceived danger a modern contraception poses to a woman’s body. When asked how she prevent unplanned pregnancy, a 37 years old mother of 4 children stated: *“I use ampicilin, because I am scared of the side effects of combination 4, Injectables and implants. I have seen people that used them and menstruated twice a month. Another person used IUD and her stomach became swollen and yet another person became lean”* (IDI Participant 4, 11 July 2016 Ekiti State). The use of ampicillin after sex is an unproven contraceptive method.

The fear of side effects of modern contraception also made some simply attempt to push out sperm immediately after sex. This view was corroborated by the response of a 25-year-old mother of two children:

(*Laughed!) “What I do is… (pause a little and continue) I usually push out the sperm immediately after sex and I douche to flush out all the sperm. I am afraid of what will happen to me should I use the family planning given to women at the health centre” (IDI participant*).

Many participants opined that modern family planning methods could result in irregular menses, bleeding, swollen stomachs, weight gain or loss, delayed return to fertility and caused vaginal to give off an odour. This fear was driven by personal experience or the experiences of friends or relatives. A 30-year-old mother of four children described her reason for not using modern contraceptives. She stated that her reason for not using modern contraception was due to her friends’ experiences. When asked about why she is not using modern contraception, she stated, “I have friends that have used pills, implants and injections and they experienced weight loss, swollen stomach, continuous bleeding, headache and fainting”. Another woman, aged 44 year who had five children stated that her younger sister’s experience influenced her decision not to use contraceptive. When asked about her reason for not using a modern contraceptive method, she responded, “I did not do modern contraception because of its side effects on my younger sister. My sister used injectables and she could not conceive for seven years after its use. One cannot give birth when you want to. A friend of mine used an implant and became lean until she had to remove it. Thus I use the withdrawal method”.

*“I will use modern contraceptive methods to limit the number of children”*.

Some women were not using any contraceptive methods or were using traditional family planning methods. They believed that modern family planning was best suited to limiting the number of children. Thus, they would only use modern family planning methods when they wanted to stop having children. A 28-year old woman who had three children corroborated this:

“*I could use an implant after I have given birth to all the children I want” (IDI participant 40, August 28, Ondo State*).

### Lack of access

One woman in Ondo state who was 30 years old mother of four children expressed her concern about limited access to family modern contraception in the community health centre in their community. According to her, “modern family planning is free but not sufficient. That is why I use the rhythm method. After they have given (contraceptives) to about four to five people, they would say to us that they (the contraceptives) are finished”.

## Discussion

This study examined the use of traditional and modern contraceptive methods among childbearing women in two southwestern Nigeria states. The study also examined the determinants of contraceptive use, reasons for non-use of — and discontinuation of contraceptives. In a bid to ensure accuracy in our estimate, we probed women with series of questions regarding child spacing and what they did to prevent pregnancy during the period they intend to space births. The findings indicated that awareness of contraception methods was almost universal and ever use of contraception was high. The findings of this study on awareness of contraceptive methods corroborated the NDHS report [12] and previous studies [11, 18, 36] that showed that awareness of family planning methods was almost universal. Improved knowledge and awareness of methods of family planning could be attributable to integration of family planning programmes into reproductive health programmes [37] in Nigeria. Women had had the opportunity to learn about the importance of child spacing and the benefits of family planning during antenatal care and child immunisation. Beyond this, access to family planning had improved due to the various efforts of government and non-government agencies. Nevertheless, many women could not accurately describe the rhythm method, female condoms and emergency contraception. These are gaps in women’s knowledge that needed to be addressed in family planning clinics.

Our study shows that 66.1% of childbearing women are currently using any methods of contraception in the two southwestern Nigeria states. Of the current users of any contraceptive methods, about half (44.3%) were using a traditional contraceptive method (withdrawal, periodic abstinence and standard day/rhythm, and lactation amenorrhea). The findings of this study indicated that the majority of women practiced a form of contraception in these two South Western Nigerian states. The qualitative findings corroborate the quantitative findings and clearly indicate that most women practised a family planning method. Similar to our study, a contraceptive use prevalence of over 50 to 70% was reported in surveys conducted in southwestern Nigeria communities [14–19]. However, these studies differ from the current study in that they were conducted among specific groups of women, such as; women attending antenatal clinics and women residing in certain small communities of southwestern Nigeria. The level of condom use in the present study is similar to surveys conducted in the study setting [16, 18, 19].

We found a high rate of use of traditional family planning methods contrary to previous studies [16, 18, 19]. The previous studies differ from this present study in that they were conducted among women attending antenatal clinic and among women residing in rural settings. The plausible reason for the high rate of traditional family planning method found in our study may be due to our willingness to probe. A study conducted in Ekiti and Ondo states shows that 46.2% of women did not disclose their method of contraceptive [19]. Researchers need to take note that discussion about contraception in cultural sensitive environments such as sub-Saharan Africa required better preparation and understanding of the cultural context. An ability to probe was non-negotiable in this context and some participants might still not have revealed the method they practiced, even when probed persistently. Some participants simply stated that such discussion was off limits and should be between husband and wife. One plausible explanation for participants’ refusing to answer or answering negatively was that women sometimes misunderstood the questions to mean use of modern contraception. In addition, methods such as the condom, the withdrawal method and the rhythm method were not named in the Yoruba language. Therefore, having to use the local language to describe the withdrawal method, for instance, was particularly awkward for many women. Thus, they might simply have stated that they do not use any method of contraception. By focusing the discussion on child spacing and what they did to prevent pregnancy when spacing their children, we were able to elicit information on family planning from the participants. The findings of this study were important in understanding the nuances of contraceptive use in Nigeria and by extension sub-Saharan Africa. The use of traditional methods was common, as reported by Dasguta et al. [38] who clearly indicated that periodic abstinence and lactation amenorrhea were the most common postpartum contraceptive methods among women in Malawi and as soon as women resumed sexual intercourse, the use of contraceptive rose to as high as 77%. The findings of the qualitative study indicate that some women even utilise some unconventional contraceptive methods to prevent pregnancy.

The quantitative finding indicates that urban area of residence was associated with contraceptive use, which is consistent with previous studies. One plausible explanation for this could be that urban areas still maintained urban privilege in allocation of family planning resources. The finding of the qualitative study corroborates this assumption. Complaints about lack of access to contraceptive methods were only from rural areas as found in the qualitative study. Thus, efforts to encourage optimal use of contraceptive methods should be intensified in rural areas.

The findings from the qualitative study shed light on the main reasons for non-use of contraceptive methods in the study settings. Fears of modern contraceptive side effects, postponement of contraceptive use and lack of access were among the reasons for non-use of contraceptives, which is consistent with previous studies [9, 10, 18, 39–41]. The narratives of women in the study setting suggest that fear of modern contraceptive side effects is the main reason for not using modern contraceptive. Many women rely on less effective (traditional methods) and some unproven contraceptive methods [21, 39] due to fear of modern contraceptives’ side effects. However, only a few women discontinued contraceptive due to experience of side effects. This finding thus illustrates that empowering women with adequate information about the effectiveness of each contraceptive method, mechanism of action and side effects would enable women to make informed choices. Providers should provide client-focused, quality, contraceptives service that respects the confidentiality of women. Counselling women about contraceptive side effects would be crucial to allay the fears they have about contraceptive side effects.

### Study limitation

Self-reporting of use and non-use of contraception was a limitation of this study, which could have led to possible underestimating of levels of contraceptive use. To minimise the impact of this limitation, the researcher trained the research assistants on methods of eliciting sensitive information. In addition, the probing technique helped to mitigate this limitation. This study was conducted in two of the six states in southwestern Nigeria, as such; the findings may not be generalizable to the whole of southwestern Nigeria.

## Conclusion

Our study suggests that use of any method of contraception is high in the study setting; however, use of modern contraceptive methods (known to be more effective) is low. Many women depend on less effective traditional contraceptive methods due to fear of side effects of modern contraception. The findings of this study confirm the study assumption that the rate of traditional contraceptive use may have been underestimated in previous studies. Eliciting information on the use of contraception —a culturally sensitive issue—in sub-Saharan Africa will require probing skills to ensure accurate data. The contraceptive calendar developed by the Demographic and Health Survey could be an important probing tool for eliciting accurate contraceptive information in sub-Saharan Africa.

## Abbreviations

IDI: In-depth interview
IUD: Intrauterine device
NDHS: Nigeria demographic and health survey
